# Characteristics and Treatment Outcomes of “Transfer-Out” Pulmonary Tuberculosis Patients in Gondar, Ethiopia

**DOI:** 10.1155/2016/1294876

**Published:** 2016-05-30

**Authors:** Tadesse Belayneh, Afework Kassu, Desalgne Tigabu, Gashaw Asmare, Sofanit Tilaye, Eveline Klinkenberg

**Affiliations:** ^1^Department of Medical Anesthesiology, CMHS, University of Gondar, P.O. Box 196, Gondar, Ethiopia; ^2^Department of Microbiology Immunology and Parasitology, CMHS, University of Gondar, P.O. Box 196, Gondar, Ethiopia; ^3^Institute of Public Health, CMHS, University of Gondar, P.O. Box 196, Gondar, Ethiopia; ^4^University of Gondar Hospital, P.O. Box 196, Gondar, Ethiopia; ^5^KNCV Tuberculosis Foundation, The Hague, Netherlands; ^6^Department of Global Health, Academic Medical Center, Amsterdam Institute for Global Health and Development, University of Amsterdam, Amsterdam, Netherlands

## Abstract

*Background*. During tuberculosis treatment, patients may transfer to continue treatment at another health facility. To ensure adherence until treatment completion, keeping track of patients is paramount. This study aimed to investigate treatment outcomes of patients who transferred out from the University of Gondar Hospital.* Methods*. This was a retrospective cohort evaluation of patients registered from 2009 to 2013. Treatment outcomes were collected from the TB registers of receiving units using a standardized data capture format.* Results*. During the study period 3,707 patients initiated treatment and 47.5% (1,760) transferred out. The study evaluated the outcome of 26% (457/1,760) patients, of whom 403 (88%) arrived in the receiving units. Overall, 79% were successfully treated and 13.8% transferred out for a second time. For all transferred-out cases, treatment outcomes were not reported to the referring unit.* Conclusion and Recommendation*. About half of the patients were transferred out to complete treatment elsewhere. Although successful treatment outcome was obtained in 79% of patients, these results were not fed back to the referring unit. Implementing a clear mechanism to communicate the arrival of and treatment outcome for transfer-out patients and appropriate patient education on treatment unit selection before treatment and during transfer-out are recommended.

## 1. Introduction

In Ethiopia, tuberculosis (TB) remains a major public health problem [1]. According to the Global TB Report 2015, Ethiopia is one of the 30 high TB burden countries and one of the 14 that have a high burden of TB, TH-HIV, and MDR-TB [2]. The directly observed treatment short course (DOTS) strategy has been implemented in the country since 1994 to control the TB epidemic [3]. Despite 100% geographical and 92% health facility coverage of the DOTS programme [3], noncompliance, low case detection, a high transfer-out rate, and deficient recording and reporting systems are still a challenge [4].

Globally, all national TB control programmes experience the challenges of intracountry movement of patients from one treatment center to another [2]. A transfer-out patient is a patient who has been transferred out at any time during treatment to continue treatment at another facility [5]. The proportion of transferred-out patients varies from one country to another. Reports indicate that in Sub-Saharan Africa it can range from 1% to 26% [6, 7]. In Ethiopia figures from 31% to 66% have previously been reported [8–10].

Treatment outcome is a key indicator in TB control and serves as a proxy of quality of TB treatment in a health care system [11]. A study in the UoGH showed that between 2003 and 2008, 42% of TB patients were transferred out to continue their treatment at nearby health units [9]. The Ethiopian Clinical and Programmatic Management of TB, TB/HIV and Leprosy guidelines [2] indicate that the outcome for transferred-out patients should be reported to the national surveillance system by the transferring unit, as this unit notified and initiated treatment for this patient. Monitoring treatment outcomes is essential in order to evaluate the effectiveness of the DOTS program [12]. This would make it possible to recognize and amend system failures before the incidence and proportion of resistant isolates rise [11]. However, there are limited studies conducted to assess the treatment outcome of PTB patients who are transferred out. Moreover, in view of the large number of transferred out patients in UoGH, it is important to know what their final treatment outcomes are and whether they are available at the transferring facility for them to be captured in the TB surveillance system. Therefore, this study was conducted to assess the TB treatment outcomes of transferred-out PTB patients who were registered and started treatment at UoGH.

## 2. Methods

### 2.1. Study Area

UoGH is a tertiary hospital serving the population of Gondar town and the remote hilly areas of northwest Ethiopia. The hospital has a DOTS clinic that has been operational since 2000 [13]. Around 740 TB patients are diagnosed and treated according to the national guidelines annually. The primary aim of the study was to evaluate treatment outcomes of PTB patients who had been transferred out from UoGH and who had arrived at their receiving centers. For logistic and resource reasons, only centers which were within a 180 km radius of Gondar town were included; these were fifteen centers.

### 2.2. Study Design and Data Collection

A retrospective analysis of the profile of transferred-out PTB patients registered at DOTS clinic of UoGH in the years 2009 to 2013 was conducted. Data were collected from March to June 2014. The registration documents reviewed contained basic information, such as age, sex, and sputum status, type of TB by history, HIV status, and phase of treatment at the time of transfer. Patients whose names, father's names, age, and sex matched between the registers at the UoGH and the receiving units and were classified as “transfers-in” in the receiving units were defined as “arrivals.” Patients whose names did not appear in the receiving units were defined as “nonarrivals.” For “arrivals,” treatment outcome was collected from the TB treatment registers of the receiving centers.

### 2.3. Definitions

The treatment outcome was categorized according to National TB and Leprosy Control Program (NTLCP) guidelines [13]. These categories were being cured (finished treatment with negative bacteriology result at the end of treatment), completed treatment (finished treatment, but without bacteriology result at the end of treatment for those who are smear-positive), failure (remaining smear-positive at five months despite correct intake of medication), default (patients who interrupted their treatment for two consecutive months or more after registration), death (patients who died from any cause during the course of treatment), transfer-out (patients whose treatment results are unknown due to transfer to another health facility), and successful treatment (a patient who was cured or completed treatment).

### 2.4. Statistical Analyses and Ethical Considerations

Data were entered, cleaned, and analyzed using the statistical package SPSS for Windows, version 20. Inconsistencies in data entry were randomly checked by reentering 10% of the raw data. Frequencies and percentages were used to describe patient characteristics and treatment outcomes. The study was approved by the Institutional Review Board of the University of Gondar. In addition, official permission was secured from the Regional Health Bureau of Amhara region as well as the involved treatment units.

## 3. Results

### 3.1. Characteristics of Transferred-Out Patients

During the study period 3,707 PTB patients were registered and initiated the treatment at UoGH DOTS clinic. Among these, 47.5% (1,760/3,707) were transferred out to complete their treatment in different treatment units after his/her transfer request.

Of these patients, 25.9% (457/1,760) were transferred to the fifteen selected units and their outcome was evaluated. Most of these transferred-out patients (see Table 1) were smear-negative (76.4%), male (59.3%), and new (91.9%) and in the age group of 19–34 years (40.0%). All the transferred-out patients were offered Provider Initiated HIV Testing and Counseling (PITC) at UoGH and accepted to be tested. The TB-HIV coinfection rate was 37.9% (173/457) and 44 of those patients (27.1%) were on cotrimoxazole prophylaxis and 54 (33.3%) on antiretroviral therapy at the time of transfer. About two-thirds of the patients (309/457, 68%) were transferred out during the intensive phase. The median time between treatment initiation and transfer-out was 46 days (range 9 to 140 days).

In the TB registers of the receiving units, only 88.2% (403/457) patients were traced and classified as “arrivals.” The remaining 54 (11.8%) were classified as “nonarrivals.” Of the “nonarrivals,” 9 (16.6%) were pulmonary smear-positive and 17 (31.5%) were HIV positive (Table 1).

### 3.2. Treatment Outcomes of Transferred-Out PTB Patients

TB treatment outcomes were analyzed for the 403 “arriving” patients only (Table 2). More than three-quarters, 78.8% (318/403), of patients had a successful treatment outcome (18.8% cured and 60.0% completed treatment) while 85 (21.1%) had an unsuccessful outcome (1.7% defaulted, 4.9% died, 0.5% failed, and 13.8% transferred out). For all 457 transferred-out patients, feedback on treatment outcomes was not reported to UoGH by the receiving units.

## 4. Discussion

Monitoring treatment outcomes is essential in order to evaluate the effectiveness of the DOTS program [12]. Many patients are diagnosed and initiate treatment at large hospitals. To enhance treatment adherence, these hospitals often transfer out patients to a clinic closer to the patients' homes, which has obvious benefits for patients and their families [14, 15]. Different studies have shown that receiving care at only one health facility improves patient compliance thereby reducing the risk of default [9, 10]. Moreover, receiving care close to home is also a key factor for compliance, as when people travel far for daily/weekly DOT they are more likely to default [10, 16]. The current study showed that nearly half of the patients were transferred out after being started on treatment. Other similar studies in the same region reported proportions in the 31.4%–42.0% range [8, 9]. The proportion of transferred-out patients in the current study was slightly higher than observed at a previous study in UoGH reporting 42% of patients were transferred out [9] and another study in the region that reported 31.4% [8]. The current observed higher proportion could be due to the establishment and expansion of treatment units, securing supplies of TB medicines in several health facilities and improving management in the TB program. These all lead patients to seek continued TB treatment in the nearby institutions. The result of current study is lower compared to a study conducted at one hospital in the same region (Felege Hiwot Hospital in Bahir Dar) in which 65.6% of patients were transferred out [10].

No patient should be lost when transferred out between treatment units. However, in the current study, 12% of patients were classified as “nonarrivals” as their names could not be identified in the register of the receiving unit. This proportion of 12% was higher compared to studies conducted in Laos and Afghanistan in which 4% and 10% of patients did not arrive and continue treatment at receiving units correspondingly [14, 17]. For these “nonarrivals” it could not be ensured whether they had continued their treatment. If not they continue to spread TB, especially when they are pulmonary smear-positive. In addition, such treatment interruption could favor the development of drug resistant TB. The possible reasons for these “nonarrivals” might be self-referral to another preferred facility before reaching the destined receiving unit or they may have died or discontinued treatment. This could be linked to patients not following instructions from the health care providers or providers not communicating clearly enough with patients or a combination of both. This needs further investigation.

A high transfer-out rate often results in a lower treatment success rate as for those for whom the result is not reported back the result is listed as not-evaluated.

This could jeopardize achieving the set TB control targets. The Ethiopian National Strategic Plan (2010–2015) has set a treatment success target of 90% [1], equal to the global set target by the WHO [18]. In this study, the overall treatment success rate for PTB patients who were transferred out was 79% which is lower than both the national and the global target. Currently, Ethiopia reports a treatment success rate of 91% for new cases registered in 2012, while for previously treated cases this is 43% [1]. The study result of 79% treatment success is below this and also lower than previously reported treatment success rates in northwest Ethiopia ranging from 86% to 95% [19–21] and than Amhara region which reported a treatment success rate of 87.5% in 2014 [22].

If TB control activities function, all patients who transfer out to other units should be recorded as transfers-in and the treatment outcome results communicated back to the referring units via feedback reports and by telephone [15, 23]. In the current study, none of the transferred-in TB patients had an outcome communicated to their referral institution. This suggests that TB reporting systems might not function optimally with regard to communication on treatment outcome of transferred patients. Reasons for this were unclear but it could be due to unawareness of the need for the procedure, negligence, expecting the other to report, and/or lack of communication modalities [24]. Lack of clear guidance and communication between referring and accepting DOTS clinics requires evaluation and immediate attention. The primary DOTS clinic should have collected treatment outcome data from the accepting clinics. Efficient counseling and follow-up of transferred-out patients are mandatory to avoid poor treatment outcomes. Developing clear guidance on how to trace and report transferred-out cases is essential to avoid spillover of TB and MDR-TB [24]. Telecommunication facilities are an area in need of improvement as many of the DOT clinics lack this. Also, the reporting of transferred patients may cause double counting of patients during routine quarterly reporting to central units if both the referring and receiving facility are notifying the patient. This should be looked into.

## 5. Limitations of the Study

The current study had several limitations: Firstly, it is specific to one setting which may make it difficult to extrapolate the findings to other settings. Secondly, due to the inherent features of a retrospective study design missing data/incomplete records may have influenced the results. Thirdly, the study unfortunately did not include a control group of patients that were not transferred out for comparison purposes. Fourthly, the linking between facility records was done using name matching and not with a unique identifier. Also, TB treatment outcomes were not available for the “nonarrivals” and their exclusion might have biased the treatment success rate. Despite the above limitations, the study result provided important insights into TB control for policy makers and TB program managers and identified the need to strengthen communication for transferred-out cases to ensure the surveillance data accurately reflect the number of patients on treatment as well as treatment outcomes of all TB cases.

## 6. Conclusion and Recommendation

In conclusion, at UoGH about half of TB patients were transferred out to complete treatment elsewhere while 14% of them were transferred out for a second time. Successful treatment outcome was obtained in 79% of transferred-out patients. However these results are not fed back to the referring facilities that should formally report the outcomes for these cases. This results in underreporting of successful treatment outcomes. A clear mechanism should be implemented to communicate the arrival of and treatment outcomes for transfer-out patients to ensure all patient information is up to date for adequate monitoring of TB control activities. Appropriate patient education on treatment unit selection before treatment and during transfer-out is also recommended.

## Figures and Tables

**Table 1:** Characteristics of transferred-out PTB patients at the University of Gondar Hospital and those who arrived and did not arrive at selected receiving units, respectively, Ethiopia, 2009–2013.

Characteristics	Transfer-out (*N* = 457) number (%)	Arriving (*N* = 403) number (%)	Nonarriving (*N* = 54) number (%)
*Sex*			
Male	271 (59.3)	244 (90.0)	27 (10.0)
Female	186 (40.7)	159 (85.5)	27 (14.5)

*Age (years)*			
≤18	137 (30.0)	111 (81.1)	26 (18.9)
19–34	183 (40.0)	168 (91.8)	15 (8.2)
35–54	106 (23.2)	98 (92.5)	8 (7.5)
≥55	31 (6.8)	26 (83.9)	5 (16.1)

*Sputum status (pulmonary TB type)*			
Smear-positive	108 (23.6)	99 (91.7)	9 (8.3)
Smear-negative	349 (76.4)	304 (87.1)	45 (12.9)

*Type of TB by history*			
New	420 (91.9)	368 (87.6)	52 (12.4)
Relapse	27 (5.9)	26 (96.3)	1 (3.7)
Return after default	4 (0.9)	4 (100.0)	0 (0.0)
Failure	6 (1.3)	5 (83.3)	1 (16.7)

*HIV status*			
Negative	284 (62.1)	247 (86.9)	37 (13.1)
Positive	173 (37.9)	156 (90.2)	17 (9.8)

*Duration in which the patient transferred out after initiation of treatment*			
<4 weeks	85 (18.6)	79 (92.9)	6 (7.1)
4–12 weeks	365 (79.9)	317 (86.8)	48 (13.2)
>12 weeks	7 (1.5)	7 (100.0)	0 (0.0)

**Table 2:** Treatment outcomes of transferred PTB patients who arrived at their receiving facility in northwest Ethiopia, 2009–2013 (*N* = 403).

Characteristics	Treatment outcomes *n* (%)					
	Cured	Treatment completed	Default	Failure	Death	Transfer-out
*Age (years)*						
≤18	17 (22.4)	72 (29.8)	0 (0)	0 (0)	6 (30.0)	16 (28.6)
19–34	29 (38.2)	96 (39.7)	6 (85.7)	2 (100.0)	8 (40.0)	27 (48.2)
35–54	24 (31.6)	57 (23.6)	1 (14.3)	0 (0)	4 (20.0)	12 (21.4)
≥55	6 (7.9)	17 (7.0)	0 (0)	0 (0)	2 (10.0)	1 (1.8)

*Sex*						
Male (=244)	43 (17.6)	145 (59.4)	4 (1.6)	1 (0.4)	14 (5.7)	37 (15.2)
Female (=159)	33 (20.7)	97 (61.0)	3 (1.9)	1 (0.6)	6 (3.8)	19 (11.9)

*Sputum status (pulmonary type of TB)*						
Smear-positive	42 (42.4)	35 (35.3)	2 (2.0)	0 (0)	4 (4.0)	16 (16.2)
Smear-negative	34 (11.2)	207 (60.1)	5 (1.6)	2 (0.6)	16 (5.3)	40 (13.1)

*Categories of TB at start*						
New	66 (17.9)	223 (60.6)	6 (1.6)	2 (0.5)	19 (5.2)	52 (14.1)
Relapse	8 (30.8)	14 (53.8)	0 (0)	0 (0)	1 (3.8)	3 (11.5)
Default	1 (25.0)	2 (50.0)	0 (0)	0 (0)	0 (0)	1 (25.0)
Failure	1 (20.0)	3 (60.0)	1 (20.0)	0 (0)	0 (0)	0 (0)

*HIV status*						
Positive (=155)	23 (14.8)	96 (61.9)	1 (0.6)	1 (0.6)	11 (7.1)	23 (14.8)
Negative (=248)	53 (21.4)	146 (58.8)	6 (2.4)	1 (0.4)	9 (3.6)	33 (13.3)

*Time period between transfer-out and transfer-in (363)*						
Same to 2nd day	31 (19.3)	91 (56.8)	3 (1.8)	1 (0.6)	13 (8.1)	21 (13.1)
3rd day	35 (19.2)	110 (60.4)	4 (2.2)	1 (0.5)	5 (2.7)	27 (14.8)
≥4th day	5 (23.8)	14 (66.6)	0 (0)	0 (0)	1 (4.8)	1 (4.8)

*Phase of TB treatment at time of transfer-out*						
Intensive	59 (21.1)	163 (58.2)	5 (1.9)	1 (0.3)	14 (5.0)	38 (13.5)
Continuation	17 (13.8)	79 (64.2)	2 (1.6)	1 (0.8)	6 (4.8)	18 (14.6)

*Overall *	**76** (18.9%)	**242** (60.0%)	**7** (1.7%)	**2** (0.5%)	**20** (4.9%)	**56** (13.8%)

## Abbreviations

DOTS: Directly observed treatment short course
HIV: Human Immunodeficiency Virus
TB: Tuberculosis
UoGH: University of Gondar Hospital
WHO: World Health Organization.
